# Directional Scattering Switching from an All-Dielectric Phase Change Metasurface

**DOI:** 10.3390/nano13030496

**Published:** 2023-01-26

**Authors:** Gonzalo Santos, Maria Losurdo, Fernando Moreno, Yael Gutiérrez

**Affiliations:** 1 Group of Optics, Department of Applied Physics Faculty of Sciences, University of Cantabria, 39005 Cantabria, Spain; 2 CNR ICMATE, Corso Stati Uniti 4, I-35127 Padova, Italy; 3 Physics Department, University of Oviedo, 33007 Oviedo, Spain

**Keywords:** metasurface, reconfigurable, phase-change material, antimony triselenide, control, directionality, zero-backward, contrast

## Abstract

All-dielectric metasurfaces are a blooming field with a wide range of new applications spanning from enhanced imaging to structural color, holography, planar sensors, and directionality scattering. These devices are nanopatterned structures of sub-wavelength dimensions whose optical behavior (absorption, reflection, and transmission) is determined by the dielectric composition, dimensions, and environment. However, the functionality of these metasurfaces is fixed at the fabrication step by the geometry and optical properties of the dielectric materials, limiting their potential as active reconfigurable devices. Herein, a reconfigurable all-dielectric metasurface based on two high refractive index (HRI) materials like silicon (Si) and the phase-change chalcogenide antimony triselenide (Sb_2_Se_3_) for the control of scattered light is proposed. It consists of a 2D array of Si–Sb_2_Se_3_–Si sandwich disks embedded in a SiO_2_ matrix. The tunability of the device is provided through the amorphous-to-crystalline transition of Sb_2_Se_3_. We demonstrate that in the Sb_2_Se_3_ amorphous state, all the light can be transmitted, as it is verified using the zero-backward condition, while in the crystalline phase most of the light is reflected due to a resonance whose origin is the contribution of the electric (ED) and magnetic (MD) dipoles and the anapole (AP) of the nanodisks. By this configuration, a contrast in transmission (Δ*T*) of 0.81 at a wavelength of 980 nm by governing the phase of Sb_2_Se_3_ can be achieved.

## 1. Introduction

The interaction of electromagnetic radiation with metallic nanoparticles (NPs) is leading to breakthroughs in a wide range of research fields, including optics, health, material analysis, communications, biology, etc. [1,2,3,4]. When incident electromagnetic radiation excites a metallic NP, the free electrons of the metal tend to oscillate at the incident radiation frequency. For certain frequencies, the energy of the incident light is transferred to free electrons so that they oscillate resonantly with maximum amplitude, leading to the Localized Surface Plasmons Resonances (LSPRs) that confine and intensify the electromagnetic field in the vicinity of the NP’s surface [5,6]. The frequency of the LSPR depends on the optical properties of the NP, its size and shape, and the wavelength of the impinging light. Although metallic NPs exhibit a good response in the UV-VIS-NIR spectral range, their intrinsic Joule losses limit their applicability in many fields [7].

Dielectric nanostructures made of high refractive index (HRI) materials can also produce the localization and intensification of electromagnetic fields with negligible Joule losses [8]. For certain photon energies, confined displacement currents can lead to resonances known as whispering gallery modes (WGM) [9,10,11,12]. Differently from LSPRs in metals, which are electric resonances, HRI nanoparticles support both electric and magnetic resonances that can be independently excited by changing the light incident wavelength. Under some specific conditions, both electric and magnetic resonances can overlap in certain spectral regions, leading to coherent effects between their scattered electromagnetic fields. These effects produce interesting angular distributions of the scattered electromagnetic energy; thus, HRI nanoparticles represent a good alternative for building scattering units at the nanoscale level to control the direction and intensity of the scattered radiation [13]. When those coherent effects are predominantly between electric and magnetic dipole resonances, two scattering directionality phenomena, known as the Kerker conditions, are produced, namely the (i) zero backward (ZB) and (ii) near-zero forward [14,15,16]. 

In HRI nanostructures with spherical geometry, the magnetic dipole (MD) is always generated at larger wavelengths than the electric dipole (ED), eliminating the possibility of overlapping both resonances by tuning the geometrical parameters of the nanostructure [17,18]. Consequently, Kerker conditions are generated “off resonance”. Therefore, although the intensity ratio between the scattered light in both forward and backward directions is high, the overall scattered intensity is relatively low. This drawback can be solved by introducing non-spherical geometries with more geometrical degrees of freedom. Nanodisks offer this possibility by allowing independent tuning of both their height (*h*) and diameter (*d*). Nanodisks offer the opportunity to select the ED or the MD as first-order resonances by modifying the aspect ratio *h/d*. Consequently, for a certain aspect ratio *h/d*, an overlap of both resonances (ED and MD) can be generated, verifying an optimal zero-backward condition, i.e., the first Kerker (ZB) condition is generated “on resonance” [19]. The experimental demonstration of the zero-backward effect for single non-spherical nanoparticles has been realized by Person et al. [20]. 

HRI nanostructures have been widely employed as building blocks for all-dielectric metasurfaces [21,22,23,24,25]. The effective refractive index of the metasurface can be controlled by tuning the material composition of the nanostructures, intrinsic and extrinsic resonances, nanostructure size, and ambient conditions. The use of all-dielectric metasurfaces has been demonstrated in a wide range of applications, such as enhanced imaging, structural color, holography, and planar sensors, among others [26,27,28]. One of the most important applications is related to the control of the scattering directionality. For instance, Staude et al. [29] proposed a metasurface based on a two-dimensional (2D) array of silicon nanodisks surrounded by SiO_2_ to minimize backward scattering through the zero-backward condition. Nevertheless, the functionality of metasurfaces is fixed at the fabrication step by the geometry and optical properties of the materials, limiting their potential as actively reconfigurable devices. This disadvantage can be solved by the integration of phase change materials (PCMs) in the metasurfaces [30,31,32,33,34,35,36]. 

Through thermal or laser irradiation stimuli, PCMs can be reversibly switched between their amorphous and crystalline phases, resulting in a modulation of their refractive index in ultra-short times (of the order of ps) [37,38,39]. Therefore, the integration of PCMs in photonics gives an extra degree of freedom to tune their optical response post-fabrication [40,41]. For example, de Galarreta et al. [42] proposed a new hybrid PCM-HRI metasurface concept in which active control is achieved by embedding deeply subwavelength inclusions of a tunable PCM (in this case Ge_2_Sb_2_Te_5_, i.e., GST) within the body of high-index Si nanocylinders. In this way, they can control the spectral position of the ED and MD of the nanodisks by changing the phase of the PCM. Their goal is based on the switch of the ED resonance upon phase transition in the GST layer. Nevertheless, in their metasurface design, there is no overlap between both contributions, and therefore, the ZB is not verified.

Here we report a reconfigurable all-dielectric metasurface based on a hybrid nanostructure of silicon (Si) and PCM antimony triselenide (Sb_2_Se_3_) for the control of the directionality of the scattered radiation. In the Sb_2_Se_3_ amorphous state, all the light can be transmitted because the zero-backward condition is fulfilled. In the Sb_2_Se_3_ crystalline phase, most of the light is reflected due to a resonance whose origin is the coherent contribution of the electric dipolar (ED), magnetic dipolar (MD), and anapolar (AP) resonances. With the proposed metasurface a contrast in transmission (Δ*T*) as high as 0.81 at a wavelength λ = 980 nm can be achieved. This paper is organized as follows: Section 2 is devoted to describing the methodology used in this research; Section 3 contains information about the device design; Section 4 includes the results and discussion; and finally, Section 5 develops the conclusions of the work. 

## 2. Methods

Finite-difference time domain (FDTD) simulations have been performed using Ansys Lumerical 2022 to obtain the transmittance spectra profiles, scattering cross-sections, and distribution of the scattered electric field in the near-field regime. Non-uniform mesh settings were used in all simulations, with a mesh accuracy of 4 nm. For the study of the isolated structures, a total-field scattered field (TFSF) source was used as well as perfectly matched layers (PMLs) in all x-, y-, and z-directions. For the metasurfaces study, the plane wave was set as the source. Periodic boundary conditions were used in the *x*- and *y*-directions to simulate an infinite number of hybrid nanodisks with the same period in both directions. In the z-direction, PMLs were set at z = −4000 and z = 4000 nm. The minimum value for the shutoff was 10^−5^.

The multipolar decomposition of ED, MD, and AP resonances from the scattering cross section of the building block has been realized by the method proposed by Hinamoto and Fujii [43]. 

## 3. Device Design 

**Figure 1:**
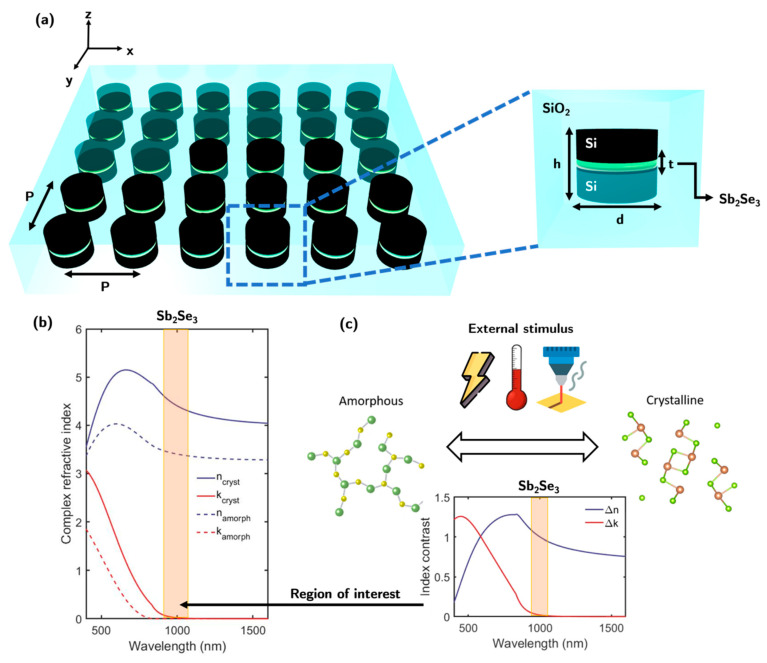
(**a**) A sketch of the reconfigurable metasurface proposed in this work. (**b**) The complex refractive index (*m* = *n* + *ik*) of Sb_2_Se_3_ amorphous and crystalline phases. (**c**) Scheme of the phase transformation (amorphous to crystalline) of Sb_2_Se_3_ through external stimulus such as, temperature, current, or pulse laser. Refractive index contrast (∆*n* = *n*_cryst_ − *n*_amorph_ and ∆*k* = *k*_cryst_ − *k*_amorph_) between crystalline and amorphous phases. The operational region of interest is shadowed in orange.

A scheme of the proposed reconfigurable metasurface is shown in Figure 1a. It consists of a 2D array of Si–Sb_2_Se_3_–Si sandwich disks embedded in a SiO_2_ matrix. The PCM thickness *t* has been fixed to 35 nm to match experimental conditions in [42,44]. The dimensions of the array (period (*P*), height (*h*), and diameter (*d*)) have been optimized to obtain the highest transmittance contrast of the metasurface between both PCM states (i.e., crystalline and amorphous). The complex refractive index (*m* = *n* + *ik*) of Sb_2_Se_3_ is represented in Figure 1b [45]. Considering that no losses (*k* = 0) are required for directional scattering, and that the refractive index contrast (∆*n* = *n*_cryst_ − *n*_amorph_) is a decreasing spectral function, the operational region of interest is located near the bandgap of the Sb_2_Se_3_ crystalline phase, which is 1.2 eV (λ_g_ = 1033 nm) [46]. Specifically, for wavelengths ranging from 975 to 1200 nm. In this region, ∆*n* ≈ 1 and ∆*k* < 0.025 are simultaneously obtained (see Figure 1c). The optical constants of crystalline silicon used for the next simulations have been obtained from Palik [47]. The effect of considering polycrystalline Si instead of crystalline Si has been considered in the supplementary information (see Figures S3 and S4).

Different external stimuli can be used for the Sb_2_Se_3_ phase transition (bias, temperature, and laser irradiation). The two most commonly used in this type of metasurface are laser irradiation [42] and the application of an external bias [48]. In order to apply an external bias, it is necessary to include a conductive material such as indium-tin oxide (ITO). The introduction of a thin layer of ITO under the high-refractive-index blocks is discussed in the supplementary information. The results shown in Section 5 differ slightly from those in Figure S1, considering this thin conductive layer. 

This Si/PCM/Si sandwich technique has already been successfully manufactured by de Galarreta et al. [42]. Additionally, the embedding of silicon nanodisks in SiO_2_ has already been realized by Staude et al. [29]. Therefore, the fabrication of the proposed metasurface is compatible with other metasurfaces already manufactured.

## 4. Results and Discussion

### 4.1. The Building Blocks

**Figure 2:**
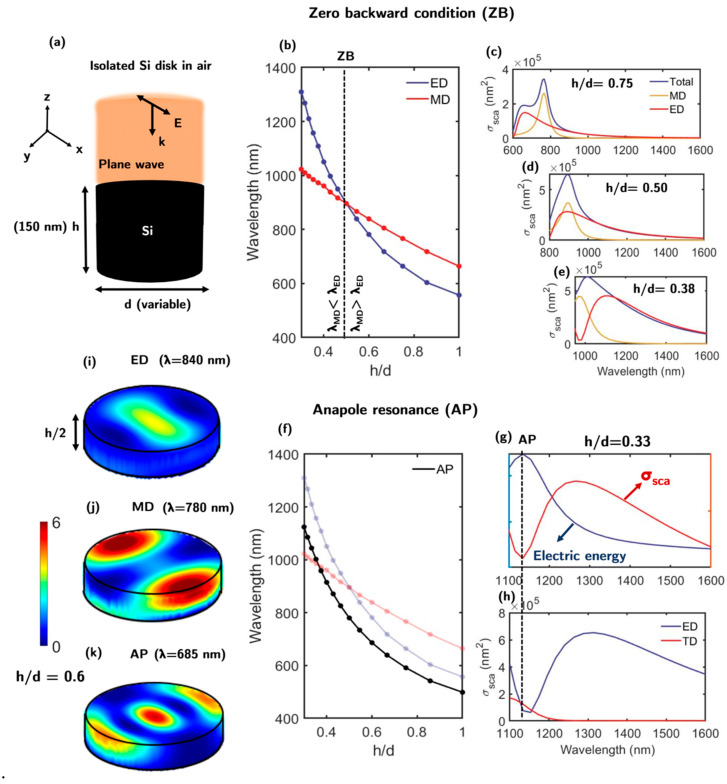
(**a**) Scheme of the isolated silicon disk in air illuminated by a plane wave in the vertical direction. (**b**) The spectral position of the electric (ED) and magnetic (MD) dipole resonances as a function of the aspect ratio *h/d*. For *h/d* = 0.5, an overlap between both dipoles is generated. (**c**–**e**) Scattering cross sections of the building block and the electric (ED) and magnetic (MD) dipole contributions for *h/d* = 0.75, 0.5, and 0.38, respectively. (**f**) Spectral positions of the ED and MD resonances along with that of the anapole (AP) resonance as a function of *h/d*. (**g**) Electric energy (blue) and scattering cross section (red) for an isolated silicon disk with an aspect ratio *h/d =* 0.33. (**h**) Scattering cross section for a ratio *h/d* = 0.33 decomposed in the ED and TD resonances. (**i**–**k**) Enhancement pattern (|E|^2^) inside the semi-disk for the ED (λ = 840 nm), MD (λ = 780 nm), and AP (λ = 685 nm) resonances, respectively, for *h/d =* 0.6 with a colorbar ranging from 0 to 6. The z position of the cutoff plane is z = 0 nm (at the center of the disk).

In order to gain a full understanding of the working principle of the proposed metasurface, the response of its building blocks is analyzed. To this end, Figure 2a shows the scheme of an isolated silicon disk (building block) in air illuminated by a plane wave propagating along the z-axis. To describe the electromagnetic response of this nanostructure when the incident wavelength approaches its dimension, a multipolar analysis of its scattering cross-section ($\sigma_{sca}$) has been performed. The response of the nanodisk is composed of three different dipolar modes: magnetic (MD), electric (ED), and toroidal (TD) [49]. The height *h* of the disks remains constant at 150 nm, while the diameter *d* is varied to analyze the magnetic and electric dipolar resonance spectral positions as a function of the disk aspect ratio *h/d*. This analysis was performed from 400 to 1600 nm to cover VIS and telecom wavelengths. Figure 2b shows the spectral position of the ED and MD as a function of *h/d*. The spectral position of ED exhibits a stronger dependence on the diameter variation as compared to the spectral shift suffered by MD. This tuning of *h/d* provides the possibility for the overlap of both resonances at a certain wavelength. In this case, both resonances overlap at an aspect ratio of *h/d* = 0.5 and a wavelength λ = 897 nm. Under these conditions, the backward scattering can be completely suppressed, satisfying the ZB condition. The scattering cross section for different aspect ratios (0.75, 0.50, and 0.38) is shown in Figure 2c–e, respectively. Depending on the value of *h/d*, either the electric or magnetic dipole can be selected as the higher resonance mode. 

Toroidal dipoles coupled with electric dipoles can produce non-radiating modes known as anapoles (AP) [50,51,52]. Through destructive interference, coherent fields emanating from toroidal and electrical dipoles cancel each other. The far field is not affected by ideal anapole excitations since they do not emit or absorb. As a result, a strong electric field is enhanced inside the dielectric nanostructure. Anapoles resonances can be detected theoretically in the spectrum by a minimum in the scattering cross section, a maximum in the electric energy, or by the intersection of the ED and TD [53,54,55,56]. The electric energy can be regarded as an essential component of the electric enhancement within the nanodisk (∫*_v_*|E|^2^ d*v*). The scattering cross section (red) and electric energy (blue) for an aspect ratio of 0.33 is represented in Figure 2g. The multipolar decomposition of ED (blue) and TD (red) from the scattering cross section and for the former aspect ratio is represented in Figure 2h. As it can be observed, the minimum of the scattering cross section, the maximum of the electric energy and the cutoff point between ED and TD dipoles matches the same wavelength. The spectral position of AP resonances for different values of *h/d* as a function of the wavelength is represented in Figure 2f. The enhancement (|E|^2^) inside the disk for ED, MD, and AP resonances is shown in Figure 2i–k, respectively. 

### 4.2. The Metasurface in Air

**Figure 3:**
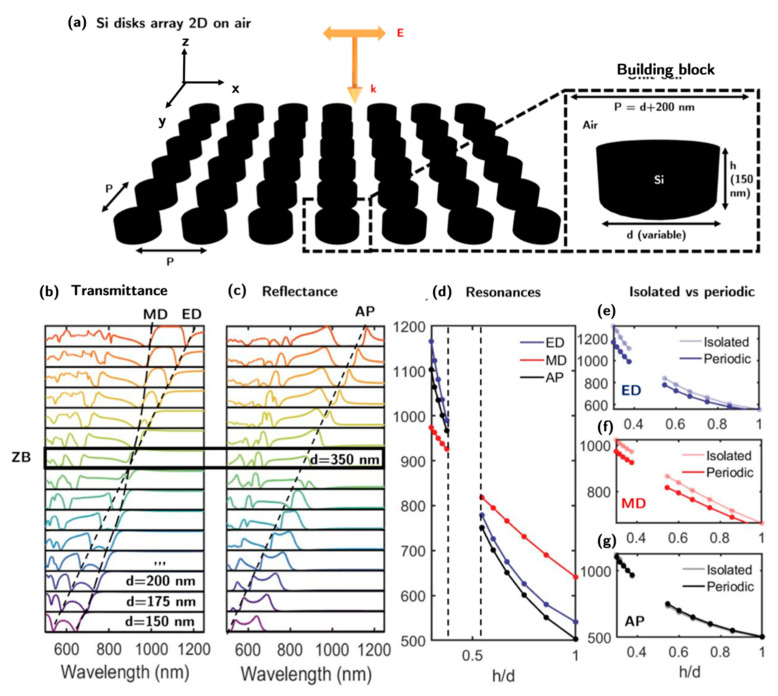
(**a**) A two-dimensional array made of silicon nanodisks in air illuminated normally by a plane wave. (**b**,**c**) Transmittance and reflectance, respectively, of a silicon disk metasurface as a function of the disk diameter, d, ranging from 150 to 500 nm. Dashed lines indicate the spectral positions of ED and MD (in the transmittance spectrum) and AP resonances (in the reflectance spectrum), respectively. (**d**) The spectral position of MD, ED, and AP resonances as a function of the aspect ratio *h/d*. There is no data available between *h/d* = 0.37 and 0.50 as it is difficult to accurately determine the spectral position of the resonances due to their spectral overlap. (**e**–**g**) Spectral positions of ED, MD, and AP resonances, respectively, as a function of *h/d*. Each case is compared with the isolated disk.

In general, metasurfaces are composed of building blocks that interact, and this interaction and the corresponding collective effects among all the blocks must be analyzed. Figure 3a shows a 2D array of silicon nanodisks surrounded by air and illuminated by a linearly polarized (along the *x*-axis) plane wave propagating along the *z*-axis. The height of the disk is fixed at *h* = 150 nm, and the diameter is varied from *d =* 150 to 500 nm. The period *P* in both directions (*x* and *y*) is the same, and it is considered the sum of *d* and 200 nm. This gap has been selected to be compatible with other experimentally demonstrated dielectric metasurfaces [29,57,58,59]. The transmittance and the reflectance of the metasurface in Figure 2a of *d* is shown in Figure 3b,c, respectively. Dashed lines mark the spectral positions of ED, MD, and AP resonances. MD and ED are associated with minimums (or maximums) in transmittance (or in reflectance). Because they do not scatter, AP have the lowest reflectance. The spectral positions of MD, ED, and AP resonances as a function of the aspect ratio *h/d* are represented in Figure 3d. In this case, there is no data available between *h/d* = 0.37 and 0.50, as it is difficult to accurately determine the spectral position due to their overlap. From the transmittance spectra in Figure 3b, the diameter and resonant wavelength for which the ZB condition is fulfilled can be estimated. Those parameters are *d* = 350 nm (i.e., as aspect ratio *h/d* = 0.43) and λ = 910 nm. Under these conditions, the transmittance is near 1, as the light scattered in the backward direction (reflected light) is mainly suppressed.

Figure 3e–g shows, respectively, the spectral positions of the ED, MD, and AP resonances. For comparison, the spectral positions of ED, MD, and AP for the isolated disk in Figure 2 are shown to highlight the effect of the periodicity in the metasurface configuration. The largest discrepancies occur for the electric dipole resonance. This tendency increases for small aspect ratios as the interaction between the disks becomes stronger. The reason lies in the fact that the ED confines the electric field around the dielectric nanostructure and not inside, making it more vulnerable to external stimuli (i.e., neighboring disks). On the contrary, the non-radiating anapolar mode confines the electric field inside it, and consequently, the spectral position of the AP is not altered by the presence of neighboring nanostructures. As the electric and magnetic dipole modes are blue-shifted by increasing interaction with neighboring disks, the contributions of the MD, ED, and AP modes near the ZB condition cannot be easily discriminated due to the proximity of the three resonances. This is the reason for the plotting gap in Figure 3d.

It is worth noting that at normal incidence, the electromagnetic response of the metasurface does not depend on the polarization of the incident beam due to the weak interaction between neighboring disks (see supplementary information, Figure S2). 

### 4.3. The Reconfigurable Metasurface in SiO_2_

**Figure 4:**
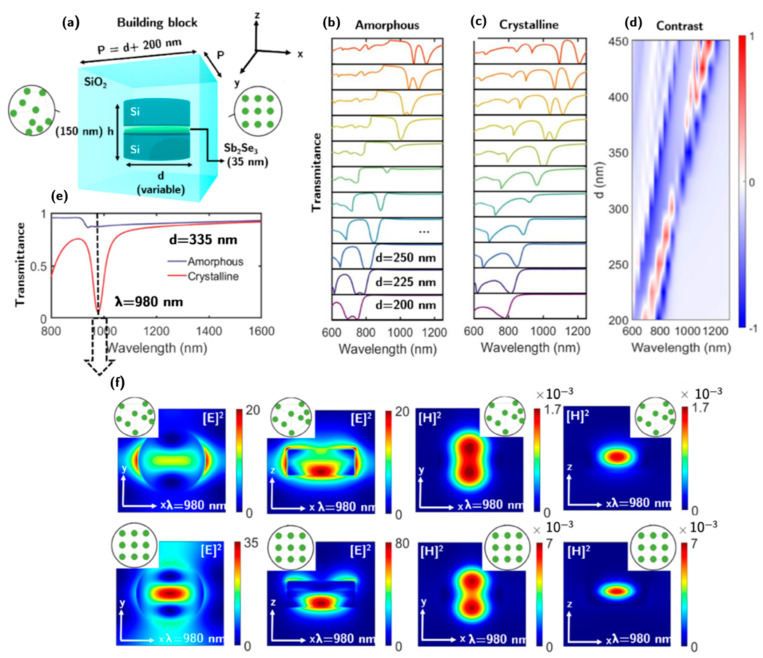
(**a**) The building block of the proposed reconfigurable metasurface. It consists of a sandwich-shaped Si–Sb_2_Se_3_–Si disk embedded in a SiO_2_ medium. (**b**,**c**) Transmittance spectra of the metasurface for the Sb_2_Se_3_ amorphous and crystalline phases, respectively, for disk diameters ranging from 200 to 450 nm. (**d**) Contrast in transmission (∆*T)* between the crystalline and amorphous phases. (**e**) Transmission spectra for amorphous and crystalline phases for a diameter of 335 nm (i.e., an aspect ratio of *h/d* = 0.45). The dashed line at 980 nm indicates the transition between the zero-backward (amorphous) and the resonance due to the contributions of ED, MD, and AP (crystalline). (**f**) Square modulus of electric and magnetic fields in the near-field regime for different planes and both Sb_2_Se_3_ phases at λ = 980 nm.

In this section, we keep the same geometrical configuration and parameters as in the previous one (Figure 4a). The same calculation procedure has been performed for both phases of the PCM. The integration of the PCM layer in the body of the silicon nanodisk slightly modifies the results presented in the previous section for the isolated disk and the metasurface configuration in air. Sb_2_Se_3_ in its amorphous state has a lower refractive index *n* than that of silicon. Therefore, a blueshift of the resonant wavelengths is observed. For crystalline Sb_2_Se_3_, the opposite happens. For example, at 980 nm, the refractive indices of Si, c-Sb_2_Se_3_, and a-Sb_2_Se_3_ are 3.61, 3.44, and 4.45, respectively. In the operational region of interest (i.e., between lambdas 975 and 1200 nm), the refractive index contrast between both states of the PCM is ∆*n* ≈ 1 with ∆*k* < 0.025. 

The inclusion of an embedding medium reduces the optical contrast between the nanostructure material and its surroundings while redshifting the resonant wavelengths. For this reason, SiO_2_ has been chosen as it is one of the materials with the lowest refractive index while also being transparent in the operational region of interest. An embedding medium is required instead of a substrate as the same environment in the forward and backward directions to rigorously satisfy the Kerker conditions.

The transmittance, *T*, of the metasurface for the amorphous and crystalline phases of the Sb_2_Se_3_ PCM layer is represented in Figure 4b,c, respectively. The contrast in transmittance between the two is shown in Figure 4d. The highest contrast (Δ*T* = 0.81) within the operational region of interest is obtained for a diameter *d* = 335 (*h/d* = 0.45) and for an incident wavelength λ = 980 nm (see Figure 4e). An analogous simulation considering the introduction of a thin layer of ITO under the high refractive index blocks is discussed in the supplementary information (see Figure S1). Under these parameters, for the amorphous state, the ZB condition is fulfilled. For the crystalline phase, a resonance is generated by the overlapping of the ED, MD, and AP resonances that suppresses the transmittance. The square modulus of electric and magnetic fields in the near-field regime for different planes and for both phases at λ = 980 nm are shown in Figure 4f. In the first column, the square modulus of the electric field is represented in the x-y plane for the amorphous and crystalline phases, respectively. For the amorphous, the radiation pattern is given by the interaction between the ED and MD as it comes out of the coherent superposition of both resonances. For the crystalline phase, the radiation pattern is a contribution from the ED, MD, and AP. The near-field electric map is predominantly characterized by the anapolar pattern as it is the mode with a higher field enhancement inside the nanostructure. 

## 5. Conclusions

A reconfigurable all-dielectric metasurface using a 2D array of building blocks made of sandwich-shaped Si–Sb_2_Se_3_–Si disks implanted in SiO_2_ has been proposed. The electromagnetic interaction of this structure with VIS-NIR radiation has been analyzed by exact numerical calculations based on the finite-difference time-domain method. As Sb_2_Se_3_ is considered a phase change material, the transition between its phases, from amorphous to crystalline, gives the metasurface its tunability concerning its reflection and transmission properties. Because of the HRI properties of the materials constituting the building blocks of the metasurface, the directional scattering can be controlled with the proposed metasurface configuration. All the light can be transmitted in the amorphous state because the zero-backward condition holds, but most of the light is reflected in the crystalline phase due to a resonance that is caused by the interaction of the electric (ED), magnetic (MD), and anapole dipoles excited in the scattering unit. With this metasurface configuration, it is possible to create a contrast in the transmitted intensity by the metasurface greater than 80% by controlling the phase of Sb_2_Se_3_.

## Data Availability

Not applicable.

## Supplementary Materials

The following supporting information can be downloaded at: https://www.mdpi.com/article/10.3390/nano13030496/s1, Figure S1: (a) Sketch of the reconfigurable metasurface proposed in this work introducing a layer of ITO of thickness 15 nm. (b) Transmission spectra for amorphous and crystalline phases for a diameter of 335 nm, height of 150 nm (i.e., and aspect ratio of h/d = 0.45) and period 535 nm with (dash lines) and without (continuous line) considering the ITO layer; Figure S2: (a) Transmittance spectra for normal incidence and for the metasurface parameters: P = 535 nm, g = 200 nm and h = 150 nm, for the amorphous phase of the PCM and for 3 different angles of polarizations at normal incidence: 0° (blue), 30° (yellow) and 70° (red). (b) Transmittance spectra for normal incidence and for the metasurface parameters: P = 535 nm, g = 200 nm and h = 150 nm, for the amorphous phase of the PCM and for 3 different angles of polarizations at normal incidence: 0° (blue), 30° (yellow) and 70° (red); Figure S3: Real (continuous lines) and imaginary part (dashed lines) of the refractive index of crystalline silicon (blue), amorphous silicon (yellow) and polycrystalline silicon (red, 50% amorphous and 50% crystalline); Figure S4: Transmittance spectra for the metasurface parameters: P = 535 nm, g = 200 nm and h = 150 nm, for the amorphous phase of the PCM and for the crystalline (blue) and polycrystalline (red) structures of Si. (b) Transmittance spectra for the metasurface parameters: P = 535 nm, g = 200 nm and h = 150 nm, for the amorphous phase of the PCM and for the crystalline (blue) and polycrystalline (red) structures of Si. Reference [48] is cited in the supplementary material.
