# Supplementary material for: Directional Scattering Switching from an All-Dielectric Phase Change Metasurface
Source: Nanomaterials (Basel). 2023 Jan 26;13(3):496. doi: 10.3390/nano13030496 (PMC9918971; doi:10.3390/nano13030496)
Supplement: Supplementary file 1 [file nanomaterials-13-00496-s001.zip › nanomaterials-2129288-supplementary.pdf]

nanomaterials

*Article*

# Directional Scattering Switching from an All-Dielectric Phase Change Metasurface

**Gonzalo Santos [1], Maria Losurdo [2], Fernando Moreno [1,*] and Yael Gutiérrez [2,3,*]**

[1] Group of Optics. Department of Applied Physics Faculty of Sciences. University of Cantabria, 39005 Cantabria, Spain
[2] CNR ICMATE, Corso Stati Uniti 4, I-35127, Padova, Italy
[3] Physics Department, University of Oviedo, 33007 Oviedo, Spain
* Correspondence: fernando.moreno@unican.es (F.M.); gutierrezyael@uniovi.es (Y.G.)

## Effect of the integration of a conductive ITO layer

In this section, we analyze the introduction of an ITO layer with a thickness of 15 nm under the high refractive index building blocks in order to manipulate the phase of $Sb_2Se_3$ by applying an external voltage as it is described in [48]. The scheme of this metasurface is represented in Figure S1a. The transmittance of the device considering this ITO layer for both phases of the PCM and metasurface parameters: h = 150 nm, d = 335 nm and P = 535 nm is plotted in Figure S1b. As it can be observed, there are low discrepancies in the transmittance profile generated by the system when introducing a conductive layer.

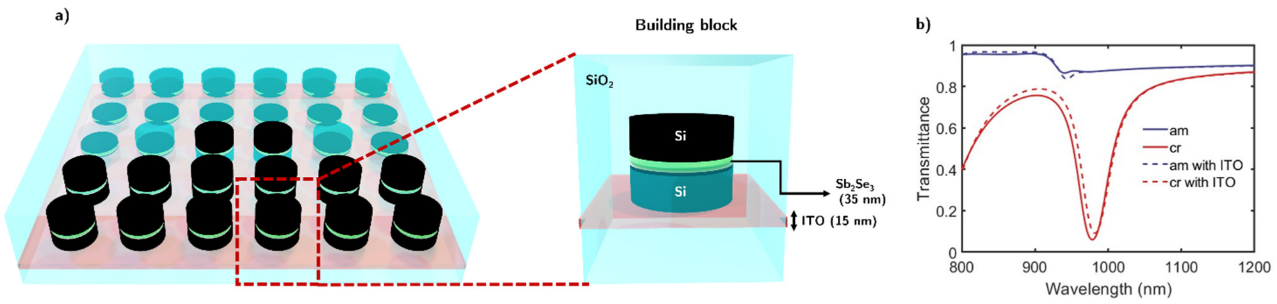


**Figure S1.** (**a**) Sketch of the reconfigurable metasurface proposed in this work introducing a layer of ITO of thickness 15 nm. (**b**) Transmission spectra for amorphous and crystalline phases for a diameter of 335 nm, height of 150 nm (i.e., and aspect ratio of $h/d$ = 0.45) and period 535 nm with (dash lines) and without (continuous line) considering the ITO layer.

## Effect of the polarization

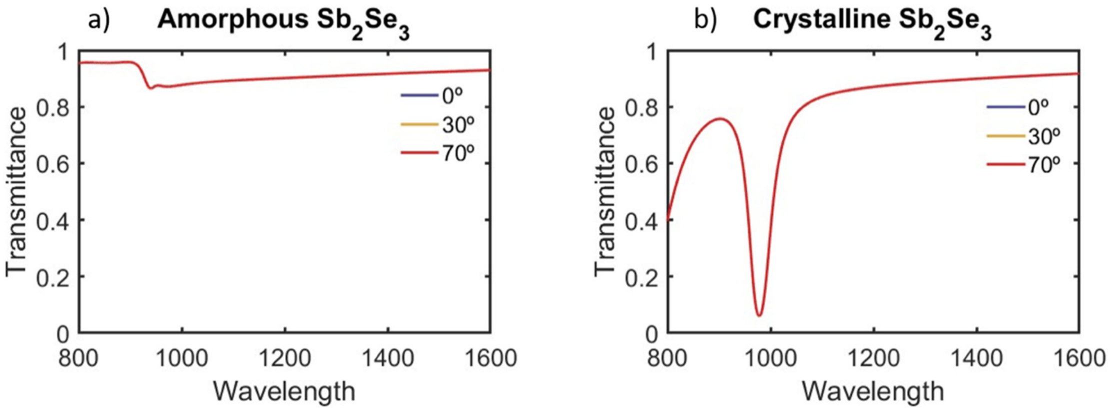


**Figure S2.** (**a**) Transmittance spectra for normal incidence and for the metasurface parameters: P = 535 nm, g = 200 nm and h = 150 nm, for the amorphous phase of the PCM and for 3 different angles

of polarizations at normal incidence: 0° (blue), 30° (yellow) and 70° (red). (**b**) Transmittance spectra for normal incidence and for the metasurface parameters: P = 535 nm, g = 200 nm and h = 150 nm, for the amorphous phase of the PCM and for 3 different angles of polarizations at normal incidence: 0° (blue), 30° (yellow) and 70° (red).

**Effect of considering polycrystalline Si**

Figure S3 shows the complex refractive index of silicon (crystalline, amorphous and polycrystalline). The optical properties of polycrystalline silicon have been obtained through Bruggeman's effective medium theory. For this, we have considered a silicon with 50% crystalline structure and 50% amorphous structure. As it can be observed, the real part of the refractive index of the three structures is very similar in the NIR, which is the region of interest of our research. The main discrepancies are in the imaginary part.

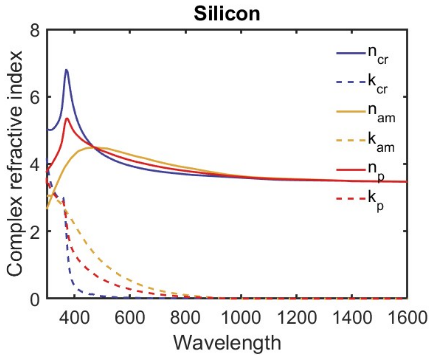


**Figure S3.** Real (continuous lines) and imaginary part (dashed lines) of the refractive index of crystalline silicon (blue), amorphous silicon (yellow) and polycrystalline silicon (red, 50% amorphous and 50% crystalline).

The simulations shown in Figure 4e in the manuscript have been again repeated but modifying the refractive index of crystalline Si to that of polycrystalline Si shown in Figure S3. The lattice parameters used are P = 535, g = 200 and h = 150. The transmittance for these parameters, for the amorphous structure of PCM and for the crystalline (blue) and polycrystalline (red) structure of Si is depicted in Figure S4a. The same procedure has been followed for the crystalline structure of PCM in Figure S4b. The discrepancies with respect to the results presented in the manuscript are very small and are due to the losses introduced in the polycrystalline structure of silicon.

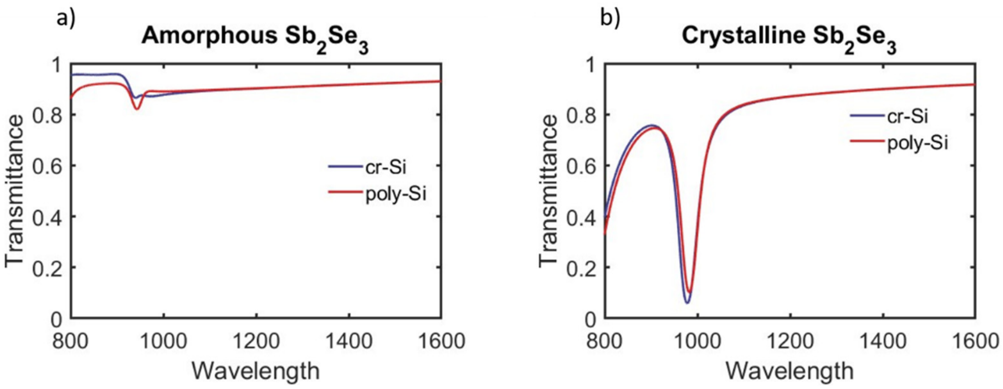


**Figure S4.** (**a**) Transmittance spectra for the metasurface parameters: P = 535 nm, g = 200 nm and h = 150 nm, for the amorphous phase of the PCM and for the crystalline (blue) and polycrystalline (red) structures of Si. (**b**) Transmittance spectra for the metasurface parameters: P = 535 nm, g = 200 nm and h = 150 nm, for the amorphous phase of the PCM and for the crystalline (blue) and polycrystalline (red) structures of Si.
